# *ESR1* and *PIK3CA* circulating tumor DNA (ctDNA) mutation status as predictive biomarkers beyond variant allele fraction (VAF) in metastatic breast cancer

**DOI:** 10.1016/j.jlb.2026.100475

**Published:** 2026-07-03

**Authors:** Nicola Fusco, Umberto Malapelle

**Affiliations:** a Division of Pathology and Somatic Molecular Diagnostics, European Institute of Oncology IRCCS, Milan, Italy; b Department of Oncology and Hemato-Oncology, University of Milan, Italy; c Department of Public Health, University of Naples Federico II, Naples, Italy

**Keywords:** Circulating tumor DNA, Variant allele fraction, ESR1, PIK3CA, Metastatic breast cancer

## Abstract

Circulating tumor DNA (ctDNA) analysis is part of the current clinical practice for the management of hormone receptor (HR)-positive/HER2-negative metastatic breast cancer, particularly for detecting actionable alterations such as *ESR1* and *PIK3CA*. Molecular testing reports commonly include variant allele fraction (VAF) as a contextual technical parameter, whose correct interpretation is increasingly important. Plasma VAF is influenced by multiple factors, including tumor fraction, metastatic site shedding, clonal architecture of the neoplasm, copy-number state, assay sensitivity, timing of sampling, and treatment pressure. In metastatic breast cancer, *ESR1* and *PIK3CA* alterations represent different evolutionary events: *PIK3CA* mutations are early, clonal, and stable; *ESR1* mutations are acquired under endocrine therapy and are often subclonal and dynamic. Consequently, VAF values across these biomarkers should be interpreted within their different biological contexts rather than as directly comparable indicators of therapeutic priority. Importantly, pivotal trials (EMERALD, PADA-1, SERENA-6, SOLAR-1, BYLieve, INAVO120, CAPItello-291) support mutation detection as the key evidence-based criterion for therapy selection. In this work, we discuss why, in metastatic breast cancer, the clinically relevant criterion for selecting *ESR1*-or *PIK3CA*-directed therapies remains the presence or absence of mutations, regardless of VAF.

## Graphical abstract

**Figure:**
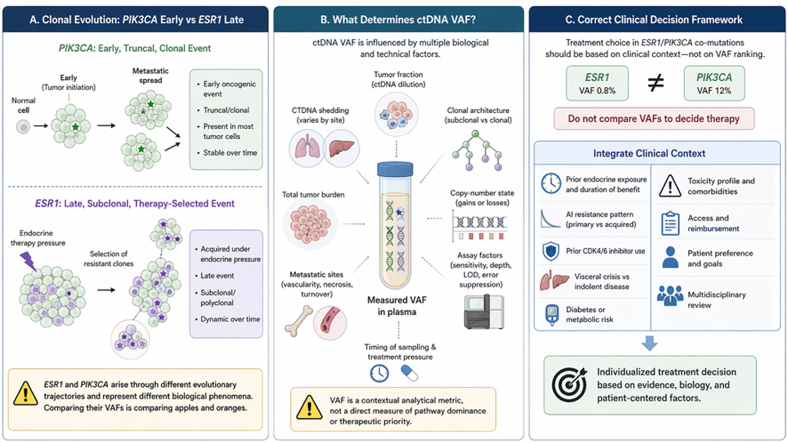

## 1 Introduction

Circulating tumor DNA (ctDNA) analysis is indispensable for the appropriate management of hormone receptor (HR)-positive/HER2-negative metastatic breast cancer, particularly for the identification of targetable genomic alterations and acquired resistance mechanisms [1,2]. For these patients, plasma-based next-generation sequencing (NGS) and highly sensitive PCR platforms are used to detect alterations involving *ESR1, PIK3CA, AKT1*, and other components of therapeutically relevant pathways [3,4]. As ctDNA testing expands, and molecular pathology reports become increasingly detailed, clinicians are confronted not only with the presence or absence of mutations, but also with additional biological and technical parameters, including variant allele fraction (VAF) [[5], [6], [7], [8]]. This adds new interpretative layers to liquid biopsy data, requiring careful multidisciplinary evaluation.

A debated issue is whether VAF values across different genes can be used to infer clinically meaningful information [9]. In breast cancer, this reasoning might translate into practical questions such as whether a patient with an *ESR1* mutation at 0.8% and *PIK3CA* mutation at 12% on ctDNA should preferentially receive a PI3K-directed therapy, or conversely whether a higher *ESR1* VAF compared to *PIK3CA* should favor the use of an oral selective estrogen receptor degrader (SERD) [10,11]. Here, we discuss the biological and clinical context of VAF interpretation in breast cancer ctDNA, emphasizing that treatment prioritization between *ESR1*-and *PIK3CA*-directed strategies should rely on clinically validated biomarkers.

## 2 Plasma VAF as a contextual analytical metric

**Fig. 1:**
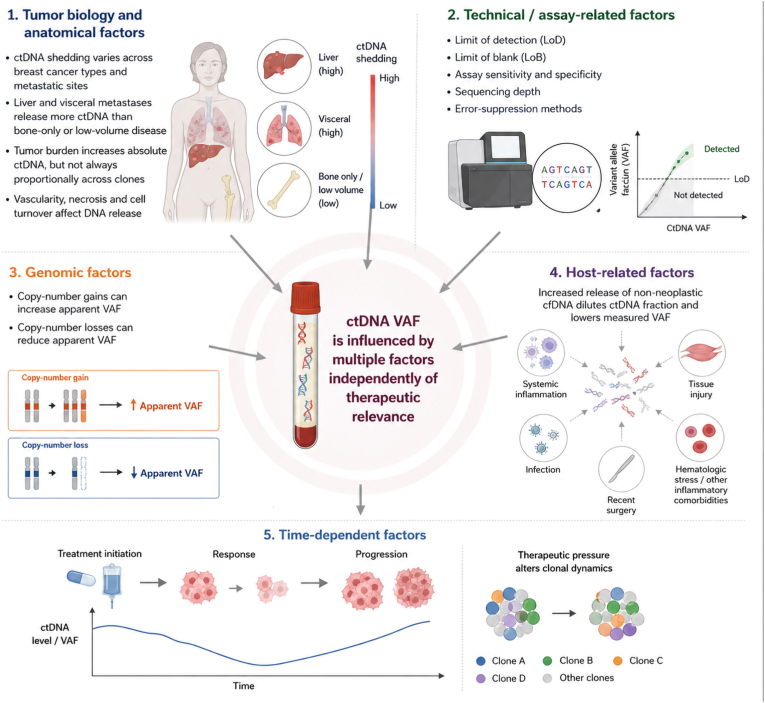
**Multifactorial determinants of circulating tumor DNA (ctDNA) variant allele frequency (VAF).** Measuring ctDNA VAF in solid tumors is influenced by several biological, anatomical, technical, host-related, and temporal variables. These include tumor burden, metastatic site distribution and shedding, assay performance, sequencing depth, error-suppression methods, copy-number status, non-neoplastic cfDNA release, timing of blood collection, and treatment-related clonal dynamics. Given these variables, cross-gene VAF comparisons are currently not validated to guide clinical treatment selection in breast cancer.

VAF in plasma-based assays is defined as the proportion of sequencing reads or amplified DNA fragments carrying a specific genomic alteration relative to the total number of analyzable cell-free DNA (cfDNA) fragments at that locus [12]. In other terms, it represents the relative abundance of a mutation within the circulating DNA compartment at the time of blood sampling [13]. In tissue samples, VAF is influenced primarily by tumor cellularity, local clonality, stromal admixture, copy-number status, and sequencing depth within a specific lesion [14]. Unlike tissue VAF, plasma VAF derives from a composite pool of cfDNA released from multiple tumor deposits and diluted by abundant non-neoplastic circulating DNA [5,15]. ctDNA therefore represents only a fraction, usually very small, of total plasma cfDNA, and the measured VAF reflects both tumor biology and systemic biological dilution [16]. Consequently, the same mutation may show markedly different VAF values in tissue and plasma, and identical plasma VAF values may arise from biologically different scenarios [17]. Several factors influence ctDNA VAF independently of therapeutic relevance (Fig. 1). The extent of ctDNA shedding varies across breast cancer types and between metastatic sites, with liver and visceral metastases generally associated with higher ctDNA release than bone-only or low-volume disease [18,19]. Total tumor burden may increase the absolute amount of ctDNA, but not always proportionally across clones. Anatomical distribution of metastases, vascularity, necrosis, and cell turnover all contribute to DNA release into the bloodstream [[20], [21], [22]]. Technical factors also substantially influence measured VAF, including limit of detection, limit of blank, assay sensitivity and specificity, sequencing depth, and error-suppression methods [23]. Beyond assay performance, genomic and host-related variables may further modify the apparent allele fraction. Copy-number gains or losses can increase or reduce the observed VAF of a given mutation, whereas conditions associated with increased release of non-neoplastic cfDNA, such as systemic inflammation, infection, recent surgery, tissue injury, hematologic stress, or other inflammatory comorbidities, may dilute the ctDNA fraction and lower the measured VAF [[24], [25], [26], [27]]. Finally, VAF is also time-dependent, as blood collection in relation to treatment initiation, response, or progression can affect ctDNA abundance, while ongoing therapeutic pressure may dynamically suppress some clones and expand others over time [[28], [29], [30]]. For these reasons, VAF should be regarded as a contextual analytical metric that describes the representation of a mutation within a specific plasma sample, rather than a direct measure of pathway dominance or oncogenic hierarchy. This aspect could be better addressed by the next generation of liquid biopsy-based assays, using multiparametric (e.g. mutations, methylations and artificial intelligence) or multilayers (DNA, RNA and protein) approaches.

## 3 Different biology of *ESR1* and *PIK3CA*

Direct comparison of *PIK3CA* and *ESR1* VAF is intrinsically challenging because these biomarkers represent distinct biological events arising at different stages of tumor evolution [31,32]. *PIK3CA* mutations are early oncogenic alterations involved in tumorigenesis and are frequently truncal or clonally maintained throughout the natural history of HR-positive/HER2-negative breast cancer [33,34]. Accordingly, they are often detectable in the primary tumor, retained in metastatic lesions, identifiable in ctDNA, and relatively stable over time [35]. By contrast, *ESR1* mutations are usually acquired under the selective pressure of endocrine therapy, particularly aromatase inhibitors, and therefore tend to emerge later during metastatic progression [36,37]. They are frequently subclonal, may occur in multiple independent resistant clones, and can change dynamically under successive treatment lines [38]. Consequently, a *PIK3CA* mutation detected at higher VAF may simply reflect its earlier clonal origin and broader representation across tumor deposits, whereas an *ESR1* mutation detected at lower VAF may represent a recently selected but clinically decisive resistance clone. This distinction supports a biologically contextual interpretation of VAF rather than a purely numerical one. Importantly, no validated VAF threshold has been shown to predict superiority of PI3K-directed therapy over oral SERDS therapy, or vice versa, in patients harboring both *PIK3CA* and *ESR1* alterations.

## 4 Multidisciplinary management of *ESR1/PIK3CA* co-mutations

The current therapeutic relevance of *ESR1* and *PIK3CA* alterations is derived from prospective clinical trials that established actionability based on mutation status (mutated vs. wild-type) as the key biomarker for treatment selection [10]. For *ESR1*, trials such as EMERALD, PADA-1, and SERENA-6 demonstrated the clinical value of ctDNA-based *ESR1* mutation detection to guide endocrine treatment adaptation, including the use of novel oral SERDs or a switch in endocrine backbone before or at disease progression [[39], [40], [41], [42], [43], [44], [45]]. For *PIK3CA*, SOLAR-1, BYLieve, and INAVO120 established the benefit of PI3K-targeted strategies in patients harboring activating *PIK3CA* mutations [46,47]. This therapeutic framework has evolved with CAPItello-291, which moved biomarker-driven treatment beyond single-gene PIK3CA targeting toward PI3K pathway-directed therapy, demonstrating the benefit of AKT inhibition in tumors harboring alterations in *PIK3CA*, *AKT1*, and/or *PTEN* [48]. None of these trials validated VAF as a biomarker for treatment selection. For this reason, in patients with concurrent ESR1 and PIK3CA mutations, ctDNA results should be discussed in a multidisciplinary setting and interpreted in the context of the overall clinical scenario [2]. The molecular pathologist contributes by clarifying tumor biology, assay performance, co-mutation patterns, and potential sources of uncertainty, while therapeutic decisions remain guided by the treating oncologist, who integrates these molecular findings with prior treatments, disease course, comorbidities, toxicity considerations, drug access, and patient preference.

## 5 Conclusions

Current evidence from prospective trials establishes the presence of actionable alterations as the validated biomarker for selecting *ESR1*-or *PIK3CA*-directed strategies. In patients harboring *ESR1* and *PIK3CA* co-mutations, multidisciplinary interpretation can integrate tumor biology, prior treatment history, disease kinetics, comorbidities, analytical limitations, and patient-centered factors. The central message is that ctDNA VAF enriches molecular interpretation, while treatment selection in HR-positive/HER2-negative metastatic breast cancer should be grounded in validated mutation status and individualized clinical assessment.

## Author contributions

N.F. and U.M. contributed equally to the conception and design of the manuscript. Both authors drafted the article, critically revised its intellectual content, approved the final version, and agree to be accountable for all aspects of the work.

## Ethical consent

Not applicable. This article does not involve human participants, human tissue, personal data, or animal research.

## Statement for studies in humans/animals

Not applicable. This article does not involve human participants, human tissue, personal data, or animal research.

## Funding sources

This work was partially supported by the Italian Ministry of Health through Ricerca Corrente 5× 1000 funds.

## Declaration of competing interest

NF: Honoraria for consulting, advisory role, speaker bureau, travel, and/or research grants from Abbvie, Alira Health, AstraZeneca, Daiichi Sankyo, Epredia, Exact Sciences, Gilead, GSK, Leica Biosystems, Lilly, Menarini Group, Merck, MSD, Novartis, Pfizer, Roche, Sakura, Sysmex, ThermoFisher, and Veracyte.

Personal/consulting or speakers' bureau fees from AstraZeneca, Amgen, Boehringer Ingelheim, Diaceutics, Diatech, Eli Lilly & Company, GlaxoSmithKline, Hedra, Janssen Biotech, Merck, Merck Sharp & Dohme, Novartis, Roche Health Solutions Inc., and ThermoFisher Scientific outside the submitted work.

UM: Editor-in-chief of the Journal of Liquid Biopsy. Speaker's Bureau for Boehringer Ingelheim, Astrazeneca, Roche, Msd, Amgen, Merck, Bms; Thermofisher, Biocartis, Menarini Stemline. Advisory Role for Boehringer Ingelheim, Msd, Amgen, Merck, BMS, Diaceutics, Roche, Eli. Lilly, Thermofisher, Janseen, Servier, Regeneron-Financial. Support that has been paid directly to my institution: Financial Support for Iis from Boehringer Ingelheim And Amgen. Recipient Of Grants/Research Supports From Astrazeneca, Thermofisher, Menarini Steamlin
